# Turpentine in Typhoid Fever

**Published:** 1890-08

**Authors:** 


### TURPENTINE IN TYPHOID FEVER.

---

Prof. H. C. Wood fears that the value of turpentine in typhoid fever is in danger of being overlooked at the present day. It was originally introduced by Dr. Geo. Wood, who taught that there are two stages of the disease in which it is particularly useful. The first stage is at the end of the second week, when the tongue becomes especially dry and glazed and the abdomen very distinctly tympanitic, or without diarrhœa. The second period is during convalescence, when perpetually recurring diarrhœa indicates failure of some of the ulcers to heal. Professor Wood states that it is his routine practice to give turpentine in every case of typhoid fever, beginning about the 12th or 15th day; and he believes that if its use were habitual, there would be fewer cases of intestinal hæmorrhage or other severe symptoms due to local lesion. It may be given with glycerin and a volatile oil made into an emulsion, in doses of 10 to 15 drops every two hours during the day-time, the patient being allowed to rest at night. The following formula is used by him:

℞. Ol. caryophylli, . . . gtt. vj.

Ol. terebinthinæ, . . . f℈iss.

Glycerinæ,

Mucil. acaciæ, . . . aa f℥ss.

Syrupi,

Aquæ, . . . aa q. s. ad. f℥iij.—M.

Sig.: Dessertspoonful as directed.

—(*Medical News*), *Satellite.*

